# Surgical Approach to Urethral Diverticulum and Urethrovaginal Fistula With Mesh Erosion

**DOI:** 10.1007/s00192-024-05787-3

**Published:** 2024-05-11

**Authors:** Alicia Smith, Lauren Burton, Saifuddin Mama

**Affiliations:** 1 Department of Obstetrics and Gynecology, Jefferson Einstein Hospital, Philadelphia, PA USA; 2 Department of Obstetrics and Gynecology, Division of Urogynecology, Cooper University Healthcare, Camden, NJ USA

**Keywords:** Pelvic surgery, Vaginal surgery, Urethrovaginal fistula, Urethral diverticulum, Mesh erosion, Urinary incontinence

## Abstract

**Introduction and hypothesis:**

This video illustrates a rare surgical case involving a urethral diverticulum, urethrovaginal fistula, and mesh erosion.

**Methods:**

We present a 58-year-old patient attending a tertiary care center with a suspected urethrovaginal fistula. Her concerns included stress urinary incontinence (SUI), recurrent urinary tract infection, and vaginal pain. The surgical history was notable for the placement of two different mesh slings during the same procedure to treat SUI. Preoperative evaluation and findings are illustrated in detail. The video uses a high-definition surgical camera to emphasize the initial intraoperative evaluation with localization of the fistula and diverticulum. We then demonstrate the approach to the dissection with the goal of ensuring complete resection of the diverticulum, fistula, and mesh, while preserving healthy tissue for subsequent closure. The utilization of unique and specialized tools for each portion of the procedure is also illustrated. A layered vaginal closure, including a Martius flap, is created to prevent recurrence.

**Results:**

The surgery was accomplished without complications.

**Conclusions:**

To our knowledge, concomitant findings of a urethral diverticulum, urethrovaginal fistula, and mesh erosion are unique in the literature. We postulate that this triad could have resulted from the mesh burden in this particular patient.

**Supplementary information:**

The online version contains supplementary material available at 10.1007/s00192-024-05787-3

## Introduction

It is estimated that 250,000 mid-urethral slings are placed annually in the USA [1]. A literature review demonstrated only four cases where a urethral diverticulum has formed after sling placement [2]. Additionally, a mid-urethral sling has a 0.3–0.8% risk of mesh erosion into the bladder or urethra [3]. The overall incidence of mesh erosion of all types varies widely throughout studies, with an average of 10.3% according to one meta-analysis from 2011 [4]. In this video we present a unique triad of a urethral diverticulum, urethrovaginal fistula, and mesh erosion. The video focuses on the complex surgical dissection via a vaginal approach. Further review of the literature illustrates two pertinent case reports related to these pathological conditions. One describes the erosion of a mid-urethral sling into a urethral diverticulum [5]. The other is a video illustrating repair of a urethral diverticulum with a superimposed fistula [6]. This video is unique because of the triad of pathological conditions that are encountered and managed with one procedure. Standard techniques to resect a urethral diverticulum were employed in this case. Similarly, standard techniques to resect a urethrovaginal fistula and to close the space were also utilized. The primary difference in the technique of this case was the concomitant resection of mesh throughout as it was encountered. The surgeon started with resection of the diverticulum and finished with resection of the fistula in order to account for any iatrogenic urethrotomies performed during the dissection. The space was then closed with multiple layers, which is common practice in fistula surgery, in order to prevent recurrence. The combination of more standardized surgical approaches leads to a unique and interesting procedure.

## Methods

We present the case of a 58-year-old patient presenting to a tertiary care center with a suspected urethrovaginal fistula. Her concerns included stress urinary incontinence, recurrent urinary tract infection, and vaginal pain. Patient-reported past medical history included interstitial cystitis, stress urinary incontinence, and intrinsic sphincter deficiency, as well as diet-controlled diabetes mellitus and chronic kidney disease stage 2. She had undergone an open supracervical hysterectomy many years prior. Most notably, she had a history of stress urinary incontinence that was treated surgically at an outside hospital with a Mini-arc sling urethropexy. During the surgery persistent incontinence was noted at 300 ml on Crede maneuver, and a second transvaginal tape was placed. The original sling was not removed. Cystoscopy and bladder overdistension were performed at the end of the procedure.

The patient presented with the majority of her work-up completed through the outside clinic, whose records were reviewed in detail. A timeline of her preoperative evaluation and those findings are illustrated in detail through this video. Important findings included a post-void residual of 15 ml, negative cystoscopy, and a negative renal ultrasound. A pelvic magnetic resonance imaging (MRI) study showed a circumferential diverticulum off the anterior inferior urethra measuring 2.9 cm × 1.6 cm × 2.1 cm. The diverticulum was noted to be contiguous with the distal vagina. Mild cystitis was also noted on MRI. Multichannel urodynamic testing was negative for stress urinary incontinence with provocation and showed a maximum urethral closure pressure of 79 cm H_2_O.

After evaluation and review of the available records, the decision was made to proceed with operative intervention. As a routine part of the consent process, the patient consented to capture of surgical photographs and video for the purposes of research and medical education. Cystourethroscopy was completed at the start of the case, which showed the presumed opening of the fistula and a portion of the diverticulum. The entire surgical case was then filmed using a high-definition surgical camera. The video emphasizes the initial intraoperative evaluation, with localization of the fistula and diverticulum. The surgeon used a variety of instruments in this portion of the case. These instruments are not required but based on the surgeon’s experience have been useful in the identification and localization of diverticula and fistulae. A Trattner Foley catheter and methylene blue dye were used to determine the margins of the urethral diverticulum. An angiocath from an arterial line kit was used to localize the fistulous tract, and was then swapped for a pediatric nasogastric tube for further manipulation.

After identification of the diverticulum and fistula tract, the surgeon opted to proceed with a midline longitudinal incision to allow for optimal exposure along the length of the urethra and to the lateral fornices. A U-shaped incision is more traditional for urethral diverticulectomy. However, given the length of the diverticulum and the need for mesh resection, the surgeon postulated that a longitudinal incision extending into the proximal vagina would provide better access. The initial dissection of the diverticulum was carried out with a scalpel and guided by methylene blue dye through the Trattner catheter. Dissection of the diverticulum was completed first and mesh was resected as it was encountered. Ultimately, two different types of mesh were encountered, one blue and one clear-white, consistent with the previously described surgical intervention. In order to fully excise the mesh, the dissection was carried out to the lateral fornices, under the pubic rami, and to the pelvic sidewall. Throughout this dissection, care was taken to preserve the periurethral and perivesical tissue so that it could later be used for closure. Dissection of the urethrovaginal fistula was completed last in order to account for iatrogenic urethrotomy. The pediatric nasogastric tube, Trattner catheter, and methylene blue dye were again used here. It was determined that the fistula communicated with the urethra, diverticulum, and vagina. Care was taken to ensure that the mucosa of the fistula was resected entirely to prevent recurrence. Discussion of a layered vaginal closure to prevent recurrence concludes the surgical portion of the video. Four layers were ultimately created. The first comprised the periurethral tissue over the urethrotomy. The second and third layers were flaps created from preserved perivesical tissue. The fourth was a Martius flap from the left labium majoris. A Martius flap is not required for closure after excision of a diverticulum or fistula [7]. It is most commonly used in cases where there is a question of tissue integrity during closure and can be fixed to support the urethra and/or bladder. In this case, the surgeon had concern over the patient’s tissue integrity at baseline and as a result of the extensive dissection required. The Martius flap was created to bolster the integrity and vascularization of the previously described closure. As the primary site of dissection was sub-urethral in the anterior vaginal wall, the Martius flap was fixed in this location. Finally, the vaginal epithelium was closed for conclusion of the case.

## Results

The surgery was ultimately accomplished without complications and the patient recovered appropriately. Bladder decompression was maintained during the initial postoperative period. As is routine to this surgeon’s practice, the patient passed an active voiding trial on postoperative day 11. At her 5-week postoperative appointment, she had rare symptoms of stress and urge urinary incontinence. Pelvic floor physical therapy referral was placed and vaginal estrogen was continued.

## Conclusion

The video presents a rare surgical case involving a urethral diverticulum, urethrovaginal fistula, and mesh erosion. A literature review yielded no other cases with these three pathological conditions occurring concomitantly. We postulate that the mesh burden from the placement of two urethrovaginal slings led to these concomitant pathological conditions. However, it is impossible to say which pathological condition was the impetus. It is also possible that the diverticulum was not recognized during the evaluation of the patient for her initial presentation of stress urinary incontinence prior to sling placement. A preexisting diverticulum could have placed the patient at an increased risk for mesh erosion and fistula formation. The aim of this video is to demonstrate the surgical approach and unique instruments that can be used in complex fistula surgery. Furthermore, this video may serve as an educational resource for future surgeons, should they encounter a similar case.

### Supplementary information

Below is the link to the electronic supplementary material.

Supplementary file1 (MP4 80996 KB)
